# The CCAAT-Binding Complex Controls Respiratory Gene Expression and Iron Homeostasis in *Candida Glabrata*

**DOI:** 10.1038/s41598-017-03750-5

**Published:** 2017-06-14

**Authors:** Antonin Thiébaut, Thierry Delaveau, Médine Benchouaia, Julia Boeri, Mathilde Garcia, Gaëlle Lelandais, Frédéric Devaux

**Affiliations:** 1 Sorbonne Universités, Université Pierre et Marie Curie (UPMC), CNRS, Institut de biologie Paris-Seine (IBPS), UMR 7238, Laboratoire de biologie computationnelle et quantitative, F-75006 Paris, France; 2 Institut de Biologie Intégrative (I2BC), CNRS UMR 9198, Université Paris-Sud, 91405 Orsay, cedex France; 3 Institut Jacques Monod (IJM), CNRS UMR 7592, Université Paris Diderot, Sorbonne Paris Cite, 75205 Paris France

## Abstract

The CCAAT-binding complex (CBC) is a heterotrimeric transcription factor which is widely conserved in eukaryotes. In the model yeast *S. cerevisiae*, CBC positively controls the expression of respiratory pathway genes. This role involves interactions with the regulatory subunit Hap4. In many pathogenic fungi, CBC interacts with the HapX regulatory subunit to control iron homeostasis. HapX is a bZIP protein which only shares with Hap4 the Hap4Like domain (Hap4L) required for its interaction with CBC. Here, we show that CBC has a dual role in the pathogenic yeast *C. glabrata*. It is required, along with Hap4, for the constitutive expression of respiratory genes and it is also essential for the iron stress response, which is mediated by the Yap5 bZIP transcription factor. Interestingly, Yap5 contains a vestigial Hap4L domain. The mutagenesis of this domain severely reduced Yap5 binding to its targets and compromised its interaction with Hap5. Hence, Yap5, like HapX in other species, acts as a CBC regulatory subunit in the regulation of iron stress response. This work reveals new aspects of iron homeostasis in *C. glabrata* and of the evolution of the role of CBC and Hap4L-bZIP proteins in this process.

## Introduction

The CCAAT-binding complex (CBC) is a heterotrimeric transcription factor which is conserved from fungi to vertebrates and plants and which specifically recognizes the CCAAT DNA motif. In yeasts, the three subunits of the CBC are called Hap2/3/5. In fungi, the Hap2/3/5 complex is sufficient to bind DNA but it requires a fourth subunit to regulate transcription of some of its target genes^1–3^. Many roles have been attributed to fungal CBC but the most extensively studied are its requirement for the regulation of respiration in *Saccharomyces cerevisiae* on one hand, and its involvement in the regulation of iron homeostasis in many other fungal species (including several human pathogens such as *Candida albicans*, *Aspergillus fumigatus* or *Cryptococcus neoformans*) on the other hand (Supplementary File S1). In the model yeast *S. cerevisiae*, the regulatory subunit Hap4 allows the CBC to activate the expression of the respiratory pathway genes in the absence of glucose^4–7^ (Supplementary File S1). In this model, *HAP4* is transcriptionally induced by non-fermentable carbon sources, while the expression and DNA binding of Hap2/3/5 are constitutive^8^. Hap4 indirectly binds DNA through its interaction with CBC. A conserved motif of 16 amino acids in the Hap4 sequence has been shown to be required for this interaction^3, 9, 10^. In the pathogenic yeast *Candida albicans* and in many other fungal species (e.g. *Aspergillus fumigatus, Aspergillus nidulans*, *Cryptococcus neoformans*, *Fusarium oxysporum*, …), the CBC plays an important role in iron homeostasis^11–19^. This role is mediated by the HapX (named Hap43 in *C. albicans*) regulatory subunit. HapX acts by repressing the expression of iron consuming genes in iron starvation conditions and by activating these genes in iron excess^11, 13, 17, 18, 20^. HapX proteins have few similarity with Hap4 except for the 16 amino acids domain (called Hap4Like or Hap4L) which is required for their interaction with Hap5^12, 18, 21, 22^. In addition, they also have a bZIP DNA binding domain similar to the one found in the oxidative stress response factor Yap1^12, 18, 21–23^. In Yap1, this domain is responsible for the specific recognition of the YRE (Yap Response Element) DNA motif (TTACTAA). The role of this domain in HapX proteins remains to be elucidated since HapX and Hap43 clearly bind CCAAT motifs indirectly through their interaction with CBC, rather than YREs^11, 20, 24^. However, HapX also contributes to the DNA binding specificity of the CBC-HapX complex in *A. nidulans* and the bZIP domain is absolutely required for the regulation of transcription by Hap43 in *C. albicans* ^18, 25^.

In *S. cerevisiae*, in contrast to *C. albicans* and *Aspergillus sp*., CBC does not seem to contribute to the global iron starvation response^26^, but its role in iron homeostasis has actually been poorly investigated in this species^27^ (discussed in refs 28, 29). A potential ortholog of Hap43/HapX, named Yap7, has been identified in *S. cerevisiae* ^18, 23^, but it is not directly involved in iron starvation or iron excess responses^21^.

In the present study, we analyzed the role of the CBC in the yeast *Candida glabrata*, a human pathogen which is evolutionary much closer to *S. cerevisiae* than to *C. albicans* ^30^. We identified the targets of Hap5 using chromatin immunoprecipitation followed by high-throughtput sequencing (ChIP-seq) and transcriptome analyses. We found that Hap5 binds many genes involved in respiration and positively controls their expression. As in *S. cerevisiae*, this function requires the Hap4 regulatory subunit. More surprisingly, we observed that Hap5 directly controls the iron stress response mediated by the Yap5 bZip transcription factor. We showed that Yap5 cannot bind the promoter sequence of *GRX4* in the absence of Hap5 and co-immunoprecipitation experiments suggested that Hap5 interacts with Yap5. Interestingly, Yap5 contains a sequence which resembles the N-terminal part of theHap4L domains of Hap4 and HapX. Full deletion or substitutions of the conserved amino acids in this sequence severely impaired Yap5 binding to its targets and its interaction with Hap5. Our results revealed a key role of CBC in the iron stress response of *C. glabrata* and identified Yap5 as a new CBC regulatory subunit.

## Results

### Hap5 targets and controls both the respiratory genes expression and the Yap5 mediated iron stress response

**Figure 1:**
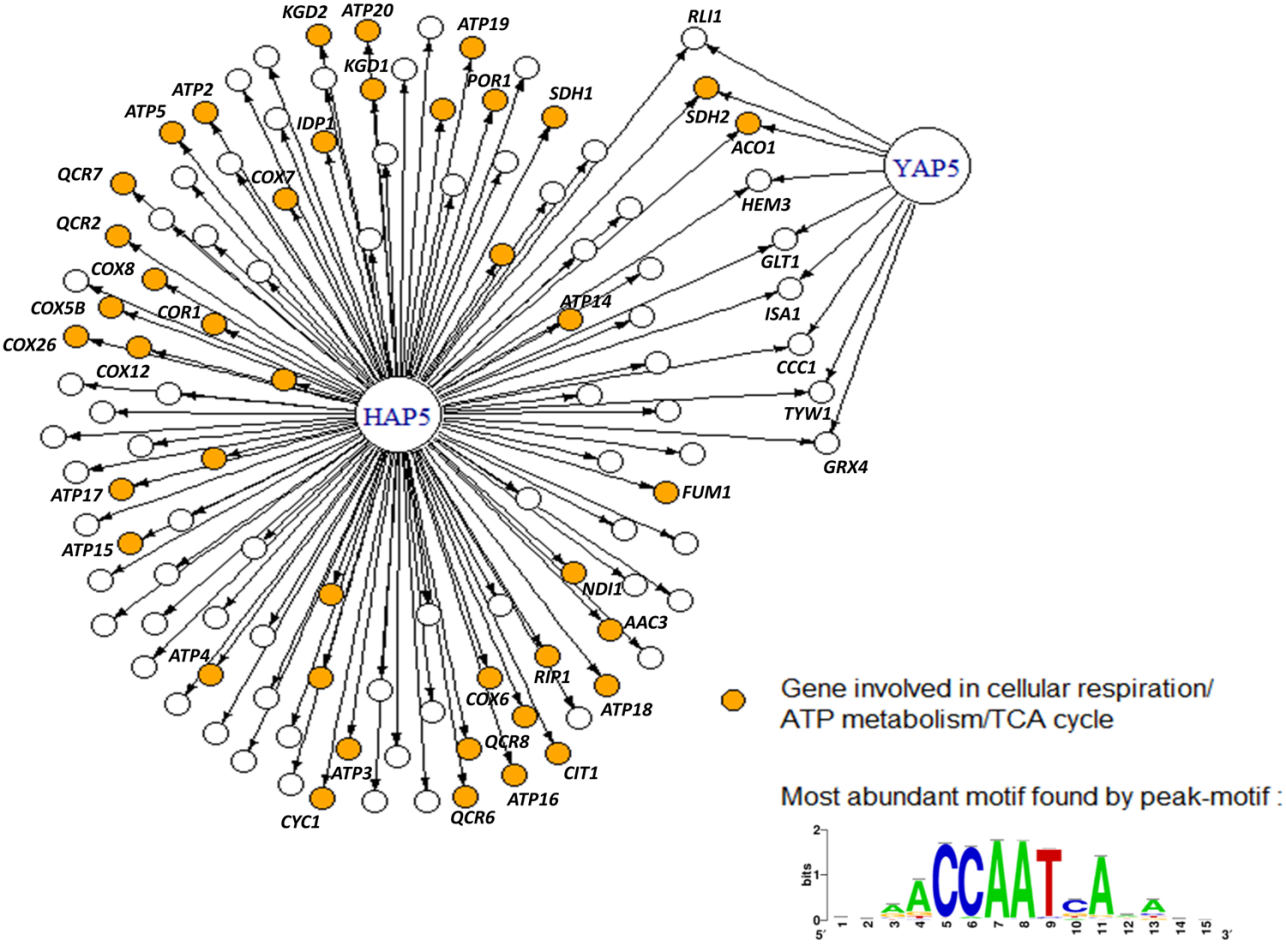
The Hap5 network in *Candida glabrata*. An arrow indicates a potential regulatory interaction based on ChIP-seq. The color of the targets indicates their belonging to respiratory pathways (yellow) or not (white). The most enriched DNA motif in ChIP peaks is represented at the bottom right. The Yap5 data are from ref. 29. The gene names indicated are those of the *S. cerevisiae* orthologs (according to the CGD database, www.candidagenome.org). For the sake of clarity, only the names of the genes which are discussed in the main text are indicated.

To identify the targets of CBC in *C. glabrata*, we analyzed the DNA binding pattern of Hap5 by ChIP-seq. We chose this subunit because it was shown in other species to be essential for the assembly, the DNA binding and the activity of CBC^31, 32^. Moreover, Hap5 is the CBC subunit which recruits Hap4 through a Hap4 recruitment domain which is present only in fungi^32^. We performed ChIP-seq on exponentially growing cells in rich glucose media. The peak calling procedure identified 113 bound promoters and 154 potential target genes (Supplementary File S2). Bioinformatic analyses using the peak motif software unambiguously identified the CCAAT box as the most enriched motif in the ChIP peaks, being present in 85% of them (Fig. 1, Supplementary File S2). Gene Ontology analyses of the list of potential target genes showed a strong enrichment in genes encoding proteins involved in cellular respiration (p = 1.6 × 10^−23^), ATP metabolism (p = 6.1 × 10^−29^), oxido-reduction processes (p = 1.7 × 10^−20^) and TCA cycle (p = 6.2 × 10^−10^). Half of the targeted promoters were associated to a gene encoding a mitochondrial protein (p = 3.4 × 10^−18^). These included genes encoding subunits of the respiratory complexes (*COX*, *ATP* and *QCR* genes, *COR1*, …), mitochondrial transporters (*AAC3*, *POR1*, …) and enzymes of the TCA cycle (*ACO1*, *SDH2*, *KGD1*, *KGD2*, …) (Fig. 1).

Another remarkable feature of the ChIP results is that all the targets previously identified for Yap5^29^ were also bound by Hap5 (Fig. 1). These correspond to 9 genes induced by Yap5 in iron excess conditions and encoding the glutaredoxin Grx4, the iron–sulfur cluster containing enzymes Aco1, Tyw1, Sdh2, Rli1 and Glt1, the iron-sulfur cluster maturation enzyme Isa1, the heme biosynthetic enzyme Hem3 and the iron vacuolar transporter Ccc1.

**Figure 2:**
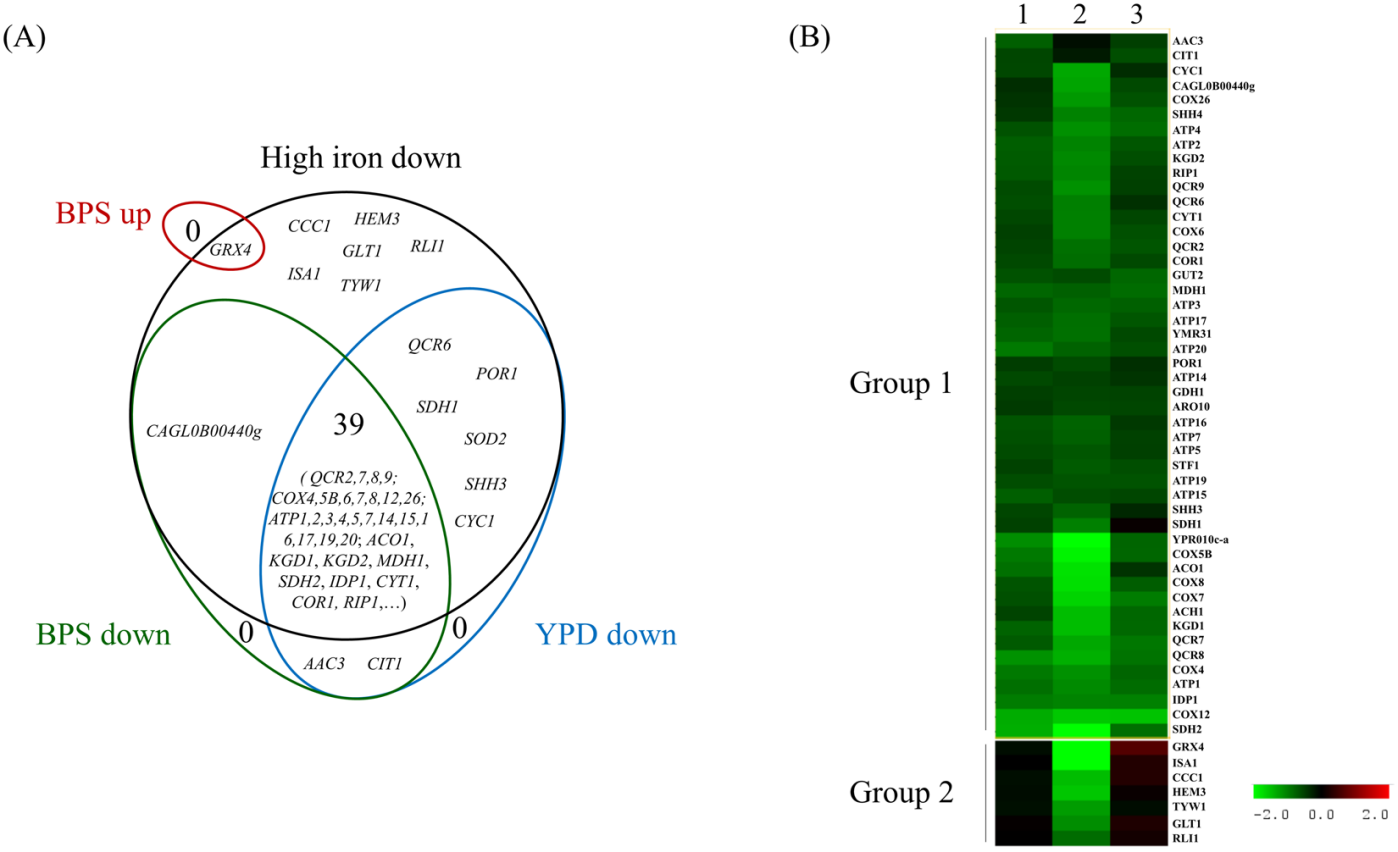
Transcriptome analyses of Hap5 impact on gene expression. The wild type and *hap5Δ* strains were grown in three different conditions (rich media, iron excess or iron starvation) and their transcriptomes were compared using microarrays. (**A**) Venn diagram representing the overlaps between the lists of Hap5 ChIP targets being significantly down regulated compared with wild type in YPD (blue line), BPS (green line) or iron excess (black line). The red line includes the only Hap5 ChIP target (*GRX4*) which was significantly up-regulated upon BPS treatment. The gene names are from the *S. cerevisiae* orthologs, when available. (**B**) Eisengram of the expression profiles of the genes from the Venn diagram. The values used are log2 of hap5Δ/wild type expression ratios. The color scale is indicated. The conditions used are **1**: YPD; **2**: iron excess (2 mM FeSO4 for 30 minutes); **3**: iron starvation (0.5 mM BPS for 30 minutes).

To measure the impact of Hap5 on the expression of its targets, we performed transcriptome analyses comparing gene expression between a *hap5Δ* mutant and wild type cells. Because our ChIP-seq results showed an interaction between Hap5 and the targets of Yap5, we performed these transcriptome experiments on cells grown in three different conditions: standard rich media, iron excess and iron starvation. Among the 113 target promoters identified by ChIP-seq, 55 showed altered expression of one of the corresponding gene in at least one of the three tested growth conditions (Fig. 2A). Two main types of targets were distinguished based on their expression profiles (Fig. 2B). The first one included 48 genes which expression was diminished in the mutant in at least two of the three tested conditions (Fig. 2). Among them, 39 showed lower expression in all three conditions (Fig. 2A). This group of « constitutively » affected targets included mainly genes encoding proteins related to respiration and mitochondrial activity. The second group included 7 genes which expression was specifically impaired in iron excess conditions (Fig. 2). These genes were all previously shown to be regulated by Yap5^29^. In this group, *GRX4* has a special status since its expression is down regulated in the Hap5 mutant in iron excess conditions but up-regulated in the same mutant upon iron starvation (Fig. 2A). Actually, this *GRX4* expression pattern is exactly the same as the one previously described for *yap5Δ* mutants^29^. ChIP-Q-PCR analyses showed that the binding of Hap5 to *GRX4* was constitutive and independent of iron availability (Supplementary File S3), as previously described for Yap5^29^.

### Hap5 cooperates with Hap4 in the regulation of respiratory genes expression and with Yap5 in the iron excess response

**Figure 3:**
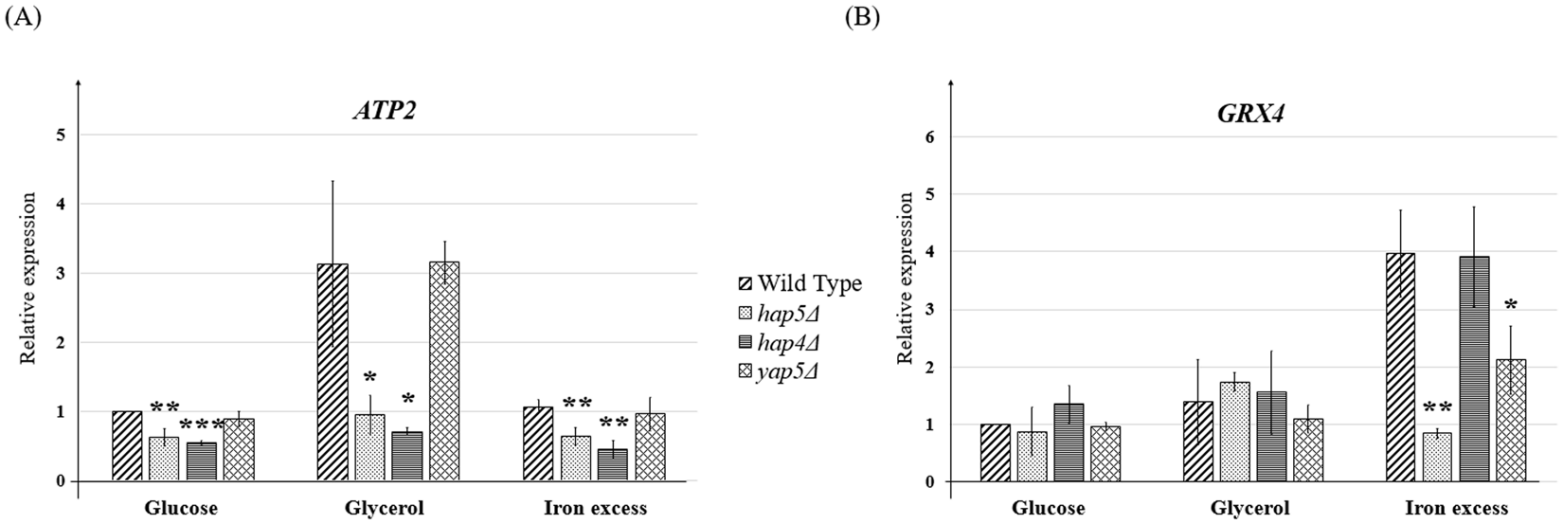
Analyses of the impact of Hap5, Hap4 and Yap5 on *ATP2* and *GRX4*. The relative expression of *ATP2* (**A**) and *GRX4* (**B**) was measured by Q-RTPCR in wild type, *hap5Δ*, *hap4Δ* and *yap5Δ* strains grown in glucose, glycerol or iron excess. The values represent the expression levels of the *ATP2* or *GRX4* genes relative to *ACT1* (used as an internal control) and to the wild type grown in glucose. The experiments were performed three times on biologically independent samples. Error bars represent the pearson standard deviation. A t-test was performed to compare, for each growth condition, the mutants to the corresponding wild type. The results of the test are indicated by the stars as follows *p < 0.05, **p < 0.01, ***p < 0.001.

Our ChIP-seq and transcriptome experiments demonstrated a dual role for Hap5 in the constitutive expression of respiratory genes on one hand, and in the iron excess activation of Yap5 targets on the other hand. The first role is consistent with what was previously shown for CBC in *S. cerevisiae*. In this species, Hap5 requires the regulatory subunit Hap4 to control the expression of respiratory genes in the absence of glucose^2, 5–7^. To clarify the potential interplays between Hap5, Hap4 and Yap5 in *C. glabrata*, we performed Q-PCR analyses of *ATP2* (group 1 in Fig. 2B) and *GRX4* (group 2 in Fig. 2B) expression in wild type, *hap5Δ*, *hap4Δ* and *yap5Δ* cells grown either in glucose, glycerol (a non-fermentable carbon source) or iron excess. As observed in our transcriptome analyses, *ATP2* levels are decreased in the *hap5Δ* mutant in all three growth conditions examined. Remarkably, the same pattern was observed for the *hap4Δ* mutant, while *ATP2* levels were independent on the presence of *YAP5* (Fig. 3A). Similar results were obtained for another respiratory gene (namely *COX12*, Supplementary File S4). In contrast, the basal levels of *GRX4* remained unaffected in all three mutants but its induction by iron excess was totally abolished in the *hap5Δ* and *yap5Δ* mutants, while being unaffected by the deletion of *HAP4* (Fig. 3B).

To further investigate the relationships between Hap5 and Yap5, we compared the Hap5 and Yap5 ChIP-peak positions and sequences. We observed that the Yap5 and Hap5 ChIP peaks were at the same locus. Moreover, seven out of nine Yap5-Hap5 ChIP targets had both a YRE motif and a CCAAT box in their Yap5 and Hap5 peaks (Supplementary File S5). Of note, *ACO1* and *SDH2*, which contain only a CCAAT-box in their promoters, are also the only two Hap5-Yap5 targets which behaved as respiratory pathway genes and not as iron excess response genes in the transcriptome experiments presented in Fig. 2. Strikingly, the YRE and CCAAT box found in the 7 other targets were always located very close to each other, with a spacing varying from 10 to 14 base pairs between the two motifs (Supplementary File S5). A genome-wide search for co-occurrences of CCAAT-box and YRE with a spacing from 10 to 14 base pairs showed that this situation is very unusual, being present in only 28 gene promoters in *C. glabrata*. This conserved and unusual spacing between the two motifs in 7 out of 9 of the promoters which were co-regulated by Hap5 and Yap5, strongly suggested that Yap5 and Hap5 had to interact to control the expression of those genes.

**Figure 4:**
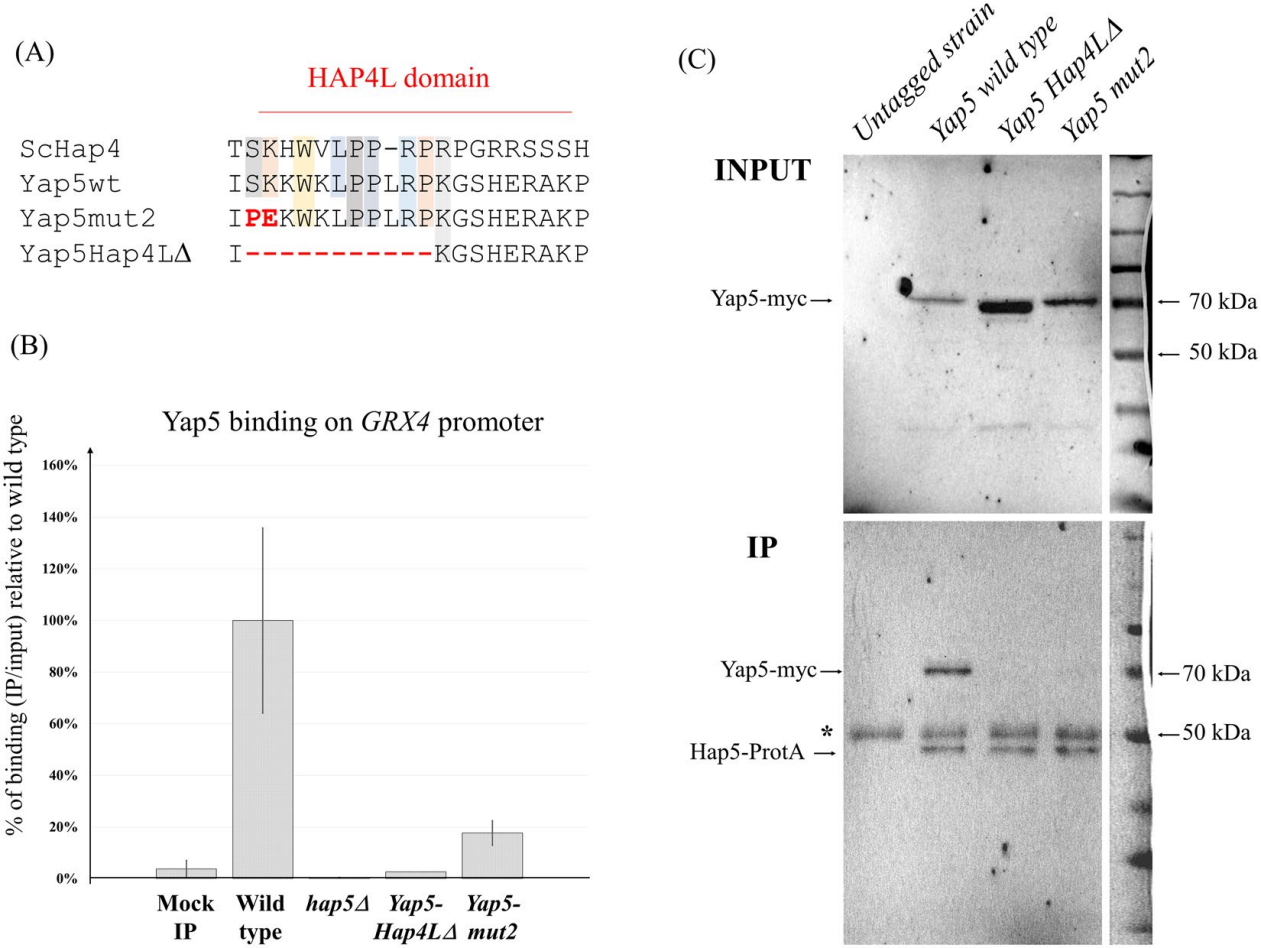
Molecular basis of the Yap5-Hap5 interaction. (**A**) Multiple alignments of the Hap4L domains of Hap4 (from *S. cerevisiae*), wild type *C. glabrata* Yap5, Yap5-Hap4LΔ and Yap5-mut2. For the latter, the substitutions are highlighted in red. (**B**) ChIP-QPCR was performed on strains expressing a myc-tagged Yap5 in presence (wild type) or absence (*hap5Δ*) of *HAP5* and on strains expressing the two different Yap5 mutant versions. All strains were grown in YPD. The values represent the IP/Input ratios of the *GRX4* promoter relative to the enrichment of the *YHB1* promoter (used as an internal control), expressed as a percentage of the enrichment obtained for the wild type Yap5. The experiments were performed twice on biologically independent samples. Error bars hence represent the standard error of the mean. (**C**) Western blot analyses of the co-immunoprecipitation experiments using Hap5-Protein A as bait and wild type or mutated versions of Yap5-myc as prey. Upper panel: input samples (INPUT), lower panel: immunoprecipitated samples (IP). Immunoblotting was performed with a mouse anti-myc antibody (Roche). The Yap5 protein is fused to 13 c-Myc epitopes and the corresponding band is expected at 65 kDa. The Hap5-Protein A fusion is expected at 45 kDa and is also detected by the anti-myc antibody (although with a low affinity), because Protein A non-specifically interacts with IgG. Note the similar intensity of the Hap5-ProtA bands in the IP, which indicates that the IP efficiency was equivalent from one lane to another. The star indicates the 50 kDa band corresponding to the large chain of the anti-Mouse antibodies used for the IP. The co-immunoprecipitation experiment was performed twice on biologically independent samples and gave consistent results. The ladder on the right was copied and pasted from the white light image of the membrane. Immunoblotting of the same membranes with rabbit IgG-HRP polyclonal antibody (PAP; code Z0113; Dako), which has a high affinity for Protein A, can be found in Supplementary File S7.

We next performed ChIP analyses of Yap5 binding to *GRX4* in wild type and *hap5Δ* cells (Fig. 4). We observed that the absence of Hap5 totally abolished Yap5 binding to its target. In a previous work, we noticed that Yap5 contains a degenerated but still recognizable Hap4L motif just upstream of its bZIP domain^21^. This motif corresponds to the N-terminal half of the canonical Hap4L domain (Fig. 4A). To assess the potential role of this sequence in the interaction between Yap5 and Hap5, we mutagenized the conserved part of the Yap5-Hap4L domain and tested by ChIP the capacity of the mutated Yap5 versions to bind *GRX4* in the presence of a wild type Hap5 (Fig. 4). First, we deleted the 11 amino acids of the conserved part of Yap5-Hap4L domain. In *C. albicans*, a similar deletion in the Hap4L-bZIP protein Hap43 was shown to abolish its capacity to regulate iron homeostasis^18^. The corresponding Yap5 mutant protein (*yap5-Hap4LΔ*) was normally expressed (Supplementary File S6) but unable to bind *GRX4* promoter (Fig. 4B). Next, we performed amino-acid substitutions at positions of the Yap5-Hap4L domain that are conserved in the Hap4 protein (S33P and K34E) (Fig. 4A). These substitutions were chosen because they were shown to abolish the activity of Hap4 in *S. cerevisiae* and therefore are likely to affect residues essential for the Hap4L-Hap5 interaction^9^. These mutations severely diminished the binding of Yap5 to *GRX4* compared to the wild type version (Fig. 4B, Supplementary File S6).

These results strongly suggested that Yap5 and Hap5 had to interact to control the expression of their common targets. To support this hypothesis, we performed co-immunoprecipitation (co-IP) experiments by tagging the chromosomic version of Hap5 with protein A and transforming the resulting strain with a plasmid bearing the wild type or the mutated versions of Yap5-myc under the control of the native *YAP5* promoter. IP samples obtained by immunoprecipitating Hap5-ProtA were then analyzed by western blot using an anti-myc antibody (Fig. 4C) or an antibody with a high affinity for Protein A (Supplementary File S7). A clear band corresponding to Yap5-myc was observed in the Hap5-ProtA IP with the wild type version of Yap5, but not with the two Yap5 versions mutated for the Hap4L domain (Fig. 4C). This difference was not due to differences in the input samples or in the IP efficiency since the input Yap5-myc signals were not lower for the mutants compared with the wild type and since the input and IP Hap5-ProtA signals were equivalent in all lanes (Fig. 4C and Supplementary File S7). This result supports the model in which Yap5 interacts with Hap5 through its Hap4L domain.

## Discussion

In this work, we identified a part of the transcriptional network associated with CBC, more particularly the Hap5 subunit, in the pathogenic yeast *C. glabrata*. As mentioned in the introduction, the main role described for CBC in the model yeast *S. cerevisiae* is the activation of respiratory pathway genes in the absence of glucose. This also involves the regulatory subunit Hap4. Very recently, it was reported that *hap5Δ* and *hap4Δ* strains have a severe growth defect in non-fermentable carbon sources in *C. glabrata*, indicating that this role may be conserved in this species^33^. Consistently with this observation, our genome-wide analyses showed that Hap5 positively controls the expression of many mitochondrial protein encoding genes, including subunits of the respiratory complexes and enzymes from the TCA cycle. We also showed that Hap4 is required for this regulation. Hence, this role in cellular respiration seems to be perfectly conserved between *S. cerevisiae* and *C. glabrata*.

**Figure 5:**
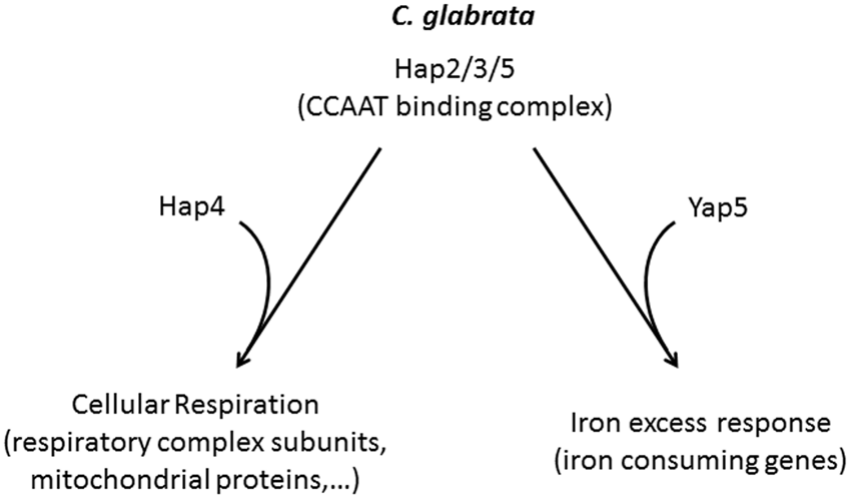
A dual role for CBC in *Candida glabrata*. CBC plays a dual role in the control of cellular respiration (together with the regulatory subunit Hap4) and of the iron stress response mediated by the Yap5 transcription factor.

More unexpectedly, we demonstrated a role of the *C. glabarata* CBC in iron homeostasis. Hap5 was found to bind the promoters of the genes of the Yap5-dependent iron stress regulon (i. e. *GRX4*, *ISA1*, *RLI1*, *HEM3*, *TYW1*, *GLT1*, *CCC1*, *ACO1* and *SDH2*) and is necessary for their induction in iron excess conditions (Figs 1, 2 and 3). Hap5 also plays a role in the negative regulation of *GRX4* in iron starved conditions (Fig. 2). This is reminiscent of the situation extensively described in *C. albicans* and many other fungal pathogens, in which the CBC plays an important role in iron starvation and iron stress responses. Hence, the CBC of *C. glabrata* seems to combine features of *S. cerevisiae* CBC (positive regulation of respiration) and *C. albicans* CBC (positive regulation of iron stress response) (Fig. 5). Interestingly enough, this is not the only example of this kind. It was recently shown that the iron starvation regulation in *C. glabrata* relies on a hybrid network which combines the “*S. cerevisiae*- like” Aft1 activity with the “*C. albicans*- like” Sef1 transcription factor^33^.

In *S. cerevisiae* and *C. glabrata*, iron stress response has been shown to be controlled by the Yap5 bZIP transcription factor^21, 34–36^. This regulation absolutely requires the direct binding of Yap5 to YRE motifs (TTACTAA) in the promoters of these genes^29, 34, 35^. We showed here that Hap5 is also necessary for this regulation to happen. More precisely, Yap5 is unable to bind and activate its targets in the absence of Hap5 (Fig. 4). Remarkably, the YRE and CCAAT motifs in the promoters of the Hap5-Yap5 iron stress regulon exhibited a conserved spacing of 10 to 14 base pairs, suggesting that the proximity of the two transcription factors on DNA is important for their activity. We previously noticed that Yap5 contains a Hap4L like domain which actually corresponds to half of the canonical Hap4L domain found in published CBC regulatory subunits such as Hap4 in *S. cerevisiae* and Hap43/HapX in pathogenic fungi^21^. Remarkably, this motif was not found in a global search for Hap4L-bZip bipartite domains^18^ and did not pass the statistical cut-off in a Hap4L search using the Pfam database^21^. Yet, partial deletion or point mutations of this domain severely reduced Yap5 binding to *GRX4* and this domain was necessary for the Yap5-Hap5 interaction detected in co-IP experiments (Fig. 4). These results suggest that, like HapX and Hap43, Yap5 needs to interact with the CBC to control iron homeostasis and that the conserved amino-acids of its truncated Hap4L domain play a key role in this interaction. In this model, Yap5 would function as a CBC regulatory subunit of a special kind, since it has its own DNA interaction which is as important for its action than its interaction with CBC and since its Hap4L domain significantly diverged from the canonical version.

**Figure 6:**
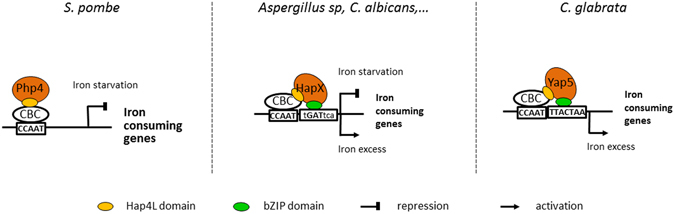
The evolution of the roles of CBC and its regulatory subunits in the control of fungal iron homeostasis. In *Schizosaccharomyces pombe*, Php4 plays an important role in the iron starvation response by repressing the iron consuming genes through its interaction with the CCAAT Binding Complex (CBC) which is mediated by its Hap4Like domain (Hap4L) (reviewed in ref. 15). In *C. glabrata*, Yap5 is a major regulator of the iron stress response which activates iron consuming genes^21, 29^. Yap5 binding to its targets requires CBC, probably by direct interaction with Hap5 through its vestigial Hap4L domain (this work). However, Yap5 also interacts directly with DNA through its bZIP domain and this interaction is essential for its regulatory activity^29, 35, 36^. Interestingly enough, the situation in filamentous ascomycetes (e.g. *Aspergillus* or *Fusarium* species) and in *C. albicans* is an intermediate between *S. pombe* and *S. cerevisiae*. HapX plays an important dual role in activating the iron stress response and in repressing the same genes in iron starvation^11, 12, 18, 20^. HapX interacts with CBC through its conserved Hap4L domain, but it also directly contributes to the binding of the CBC-HapX complex to a bipartite DNA motif, probably through its bZIP sequence^18, 20, 24, 25^.

This observation shed new interesting light on the conservation of the role of CBC in fungi and on the divergence of the associated regulatory mechanisms (Fig. 6). In the fission yeast *Schizosaccharomyces pombe*, iron homeostasis is regulated by Php4 which negatively controls the expression of iron consuming genes in iron starved cells by binding to the CBC through its HAP4L domain and by sensing iron by the intermediate of Grx4 (reviewed in ref. 15). As far as we know, Php4 does not directly bind DNA and does not contain a bZIP domain. In many pathogenic and non-pathogenic fungi (*Aspergillus* sp., *Cryptoccocus neoformans*, *Fusarium* sp., *Candida albicans*), HapX/Hap43 proteins negatively regulate iron consuming genes in iron starvation and positively regulate them in iron excess^12, 13, 16–18, 20^. They directly sense iron concentration through conserved cysteine rich motifs^20^. Gene expression regulation by HapX involves both binding to CBC and direct interaction with a DNA consensus (TGAC) which is close to a half-YRE site and which may rely on the bZIP domain of HapX^11, 20, 25^. The Yap5 functioning described here is very close to HapX, except that Yap5 binds a full YRE site^29, 34, 35^. Interestingly, Yap5 senses iron excess through a cysteine rich domain (CRD) which is very similar to the CRD of HapX^37^. Moreover, like HapX, *C. glabrata* Yap5 can act both as a repressor or an activator, depending on iron availability^29^. Then, CBC’s role in iron homeostasis is globally conserved (except that its role in iron starvation in *C. glabrata* is marginal and restricted to the repression of *GRX4*) but its mechanisms of action have diverged from a Hap4-like functioning in *S. pombe* to a fully cooperative DNA binding model in *C. glabrata*. In this model, the CBC-HapX case would be an intermediate situation, in which HapX also contribute to DNA binding specificity and affinity but in which the interaction with CBC is predominant (Fig. 6).

This model also provides keys to the answer to a long standing question, which is the basis of the Yap DNA binding specificity. Yap1, Yap2, Yap5 and Yap7 all recognize TTACTAA as their favorite consensus binding site. Yet, ChIP-seq and ChIP-chip analyses revealed that they have quite different sets of targets^29, 38^. The strong dependence of Yap5 DNA binding on the presence of both an YRE motif and of a CCAAT motif bound by Hap5 at a distance of 10–14 base pairs may partly explain its target specificity compared to the other Yap proteins, since this particular co-occurrence is very unusual in the genome of *C. glabrata*.

Additional roles of CBC have been described in fungi. For instance, in *C. albicans* and in *S. cerevisiae*, CBC has a role in the transcription activation of genes required for growth on poor nitrogen sources (e.g. *GDH1*, *GDH3* and *ASN1* in *S. cerevisiae*; *MEP2* and *SAP2* in *C. albicans*)^19, 39–44^. This activity is independent of the regulatory subunits Hap4 and Hap43^39–44^. It rather involves cooperative action between the CBC and specific DNA binding transcription factors, such as the GATA factor Gln3 in *S. cerevisiae* ^42^. Moreover, CBC has been shown to be a negative regulator of ergosterol biosynthesis and azole resistance in *Aspergillus fumigatus* ^45^, while having a positive role on *ERG9* expression in *S. cerevisiae* ^46^. Finally, CBC plays an important role in the oxidative stress response of *Aspergillus nidulans* and *Hansenula polymorpha* ^23, 47^. The overexpression of the Hap4B regulatory subunit of *H. polymorpha* (which is orthologous to the HapX proteins^48^) is able to complement the *yap1Δ* sensitivity to hydrogen peroxyde in *S. cerevisiae* ^23^. Our ChIP-seq results also suggest that some of these roles may be conserved in *C. glabrata*. Indeed, the glutamate dehydrogenase encoding gene *GDH1* and the ammonium permease encoding gene *MEP3* were targeted by Hap5 in our experiments. Also, a handful of oxidative stress response genes were bound by Hap5 in *C. glabrata* (e.g. *OYE2*, *SOD2*, *CTA1*, …). Further investigations will be required to decipher the role of Hap5 in these processes.

## Methods

### Strains and primers

The lists of the strains and primers used in this study are available in Supplementary Files S8 and S9, respectively. All the deleted strains used in this study were obtained from existing collections^21, 49, 50^ and were verified by PCR before use. The genomic myc-tagging of Hap5 was performed as described previously^51^. Briefly, myc-tagging cassette was PCR amplified from the M. Longtine’s plasmids^52^ with oligonucleotides containing homology sequences flanking the desired genomic insertion points in 5′. At least 10 micrograms of purified PCR product was used to transform HTL cells using a standard yeast transformation protocol^21^. Genotyping of the clones growing on selective media was done by PCR. The correct myc-tagging of Hap5 was verified by sequencing of the gene and western blot^21^.

The genomic TAP-tagging of Hap5 was obtained with the same strategy except that the *TAP-TRP1* cassette was amplified from the pBS1479 plasmid^53^.

For the mutagenesis of *YAP5*, we started from the pGRB2-YAP5myc plasmid^21^ and used the Quick change site-directed mutagenesis kit from Agilent, following the recommendations of the supplier. The mutant plasmids were controlled by sequencing. After transformation in *C. glabrata* using the “one-step” yeast transformation protocol^21^, the correct expression of the Yap5 mutant proteins was tested by western blot (Supplementary File S6).

### Yeast Cultures and Growth Conditions

All cultures were conducted in a rotative shaker at 30 °C. The standard growth media was YPD (Glucose 2%, yeast extract 1%, Bactopeptone 1%). For growth in non-fermentable conditions, glucose was replaced by 2% glycerol. The following stress conditions were used: 2 mM iron sulfate in CSM (2% glucose, 0.67% Yeast Nitrogen base, 0.08% Complete Synthetic Media (MP Bio)) for iron excess conditions and 0.5 mM bathophenanthroline disulfonate (BPS) in YPD for iron starvation conditions. The cells were exposed to the corresponding stress for 30 minutes. The strains harboring *HIS3* or *TRP1* selection cassettes were grown in solid or liquid CSM media without the corresponding amino acid.

### Chromatin Immunoprecipitation and High-Throughput Sequencing (ChIP-seq)

For ChIP, myc-tagged strains were grown in YPD until exponential phase (OD = 0.8). Cross-linking of the cells and ChIP were performed as described previously^54^. The parental HTL (untagged strain) was grown and processed the same way to provide the mock-IP samples. Sequencing of the IPs, Input DNAs and mock IPs samples and primary data analyses (quality controls and mapping of the reads) were performed as described previously^54^. Peak calling was performed with the bpeaks software^51^, using both the Input DNA and the mock IP as references. For peak calling using the Input DNA as reference, the bpeaks parameters were T1 = 1.9, T2 = 6, T3 = 1, T4 = 0.7. For peak calling using the Mock IP as reference, the bpeaks parameters were T1 = 1.9, T2 = 6, T3 = 1, and T4 = 0. Only the peaks that were found with both analyses were kept for further processing. These peaks were then manually checked on a genome browser^55^ to discard artefactual peaks (e.g., peaks centered on a tRNA locus, peaks perfectly overlapping a highly expressed ORF) which would have escaped the bpeaks filter. Peaks located outside of a promoter region (i.e. between convergent ORF or inside ORFs) were also discarded from the final list presented in Supplementary File S2.

### DNA Motif Enrichment analyses

DNA sequences of ChIP peaks were retrieved from their genomic locations (BED file) using the “getfasta” function from the BEDTOOLS suite^56^. These genomic sequences were used as inputs for the peak-motif tool to search for regulatory motifs^57^. An additional filtering step was added to the standard peak motif procedure to discard low complexity motifs (e.g., CCCCCCC).

The co-occurrences of YRE and CCAAT motifs in the ChIP peaks shared by Yap5 and Hap5 were confirmed by visual inspection of the peak sequences using IGV^55^. The genome-wide search of YRE-CCAAT co-occurrences with a spacing of 10–14 bp in *C. glabrata* promoters was performed using the DNA pattern search in RSAT^58^ and a dedicated R-script which automatically identified co-occurrences in promoters and measured the distance between the two motifs.

### Network Building

The ChIP peaks were assigned to genes as described previously^51^. When a peak was located in a divergent promoter (i.e., an intergenic region in between two divergent genes) the two genes were fused in one target in the network named “gene 1/gene 2”. The network was represented using the igraph R library^59^. The presence of a ChIP peak was used as the parameter to define interactions (arrows) between Hap5 and its targets. The GO criterion was used to differentially color the target promoters (Fig. 1). GO analyses were performed using the “GO term finder” tool at the CGD database, with default parameters^60^.

### Transcriptome Analyses

Knock-out and wild type strains were grown in 50 mL of YPD until exponential phase (OD = 0.8) and then stressing agents were added. After 30 min, 15 mL of each cell cultures were flash-frozen in two volumes of cold ethanol and collected by centrifugation. Total RNA was extracted, quality controlled and quantified as described previously^21^. One microgram of total RNA was used for fluorescent cDNA synthesis according to the amino-allyl protocol^21^. The cDNAs were labeled with Cy3 and Cy5 and hybridization was performed as previously described^21^. Two biologically independent experiments were performed for each condition, using dye switch. We used custom *C. glabrata* Agilent arrays in an 8 × 60 k format (array express accession number: A-MEXP-2402). After overnight hybridization and washing, the slides were scanned using a 2- micron Agilent microarray scanner. The images were analyzed using the feature extraction software (Agilent technologies) and normalized using global LOESS^61^. The mean of the biological replicates was calculated. A gene was considered as being differentially expressed if its mean absolute Log2(fold change) value was more than 0.5 and if its expression variation was considered as being statistically significant using the LIMMA package with a cut-off p-value of 0.05^62^.

### Real Time Quantitative PCR (Q-PCR) analyses

For RNA extraction, cDNA synthesis and qPCR, cells were grown in the appropriate medium until exponential phase (OD = 0.8), then stressing agents were added for 30 min when required. Cells were then snap-frozen in cold ethanol and collected by centrifugation. Cells lysis was mechanically performed with glass beads using a Fastprep®-24 bead beater (MP Biomedicals). Total RNA extraction was carried out using the RNeasy extraction kit (Qiagen) following the manufacturer’s instructions. The concentration of each sample was determined using NanoPhotometer® spectrometer (IMPLEN). For each sample, 1 μg of the total RNA were DNAse treated using Turbo DNA-free kit (Ambion). After DNAse treatment, 0.2 μg of total RNA were used to perform cDNA synthesis using Superscript II Reverse Transcriptase according to the manufacturer’s instructions (Invitrogen). The resulting cDNA were diluted to three different concentrations (1:10, 1:20 and 1:40). Quantitative PCR reactions were performed on a C1000 TM Thermalcycler (Bio-rad) with a 2X SYBR Green master mix (Promega). The qPCR reaction mixture contained 0.5 μM of each primer and 4 μL of one of the three dilutions of the cDNA. These dilutions served as triplicate for each sample. The primers used for qPCR are listed in Supplementary File S7. The relative expression for a given gene was generated by calculating the difference in the abundance between the transcripts of this gene compared to the transcripts of the *ACT1* gene, used as an endogeneous reference, based on the ΔCt method. Finally, the expression values were normalized with the expression of the studied gene in the wild type strain grown in glucose given the arbitrary value 1 (Fig. 3).

For Chromatin-immunoprecipitation followed by qPCR (ChIP-qPCR), three serial dilutions (1:4, 1:8, 1:16) of immunoprecipitated samples were simultaneously processed together with Input samples used for normalization. The primers used for qPCR are listed in Supplementary File S7. The enrichment of the *YHB1* promoter was used as an endogenous control. Q-PCR was performed as described above. The relative enrichment of a specific locus in the immunoprecipitated DNA relatively to the Input DNA and to the *YHB1* promoter enrichment was determined using the ΔΔCt method.

### Co-immunoprecipitation experiments and western blots

Co-immunoprecipitation using Hap5-Protein A as bait was performed as described^63^ except that we started from 100 mL of cell culture at OD = 0.8 in CSM-His media, that we used Dynabeads^TM^ PanMouse IgG and that all the IP sample was mixed with 2X Laemmli for western blots. Proteins contained in 15 µl of Input or IP samples were separated on 10% SDS-Polyacrylamide gel electrophoresis (SDS-PAGE). Proteins were then transferred to Whatman® Protan® BA83 nitrocellulose membrane (GE Healthcare). Immunoblotting of Yap5-myc wild type and mutant proteins were performed using 1:10000 mouse IgG Anti-cMyc (Roche) and 1:10000 anti-mouse IgG-HRP (Promega) as primary and secondary antibodies. Detection of the signals was performed using G:BOX Chemi XT4 (Syngene) following incubation with UptiLightTM HRP blot chemiluminescent ECL substrate (Interchim).

### Data availability

The ChIP-seq data can be downloaded from the GEO database (accession number: GSE91371). The complete microarray data are available at Array express database under the accession number: E-MTAB-5348.

## Electronic supplementary material

supplementary files S1 and S3 to 7

supplementary file S2

## References

[1] McNabb DS, Xing Y, Guarente L (1995). Cloning of yeast HAP5: a novel subunit of a heterotrimeric complex required for CCAAT binding. Genes Dev.

[2] Forsburg SL, Guarente L (1989). Identification and characterization of HAP4: a third component of the CCAAT-bound HAP2/HAP3 heteromer. Genes Dev.

[3] McNabb DS, Pinto I (2005). Assembly of the Hap2p/Hap3p/Hap4p/Hap5p-DNA complex in Saccharomyces cerevisiae. Eukaryot Cell.

[4] Forsburg SL, Guarente L (1989). Communication between mitochondria and the nucleus in regulation of cytochrome genes in the yeast Saccharomyces cerevisiae. Annu Rev Cell Biol.

[5] Dang VD, Valens M, Bolotin-Fukuhara M, Daignan-Fornier B (1994). A genetic screen to isolate genes regulated by the yeast CCAAT-box binding protein Hap2p. Yeast.

[6] Lascaris R (2003). Hap4p overexpression in glucose-grown Saccharomyces cerevisiae induces cells to enter a novel metabolic state. Genome Biol.

[7] Zitomer RS, Lowry CV (1992). Regulation of gene expression by oxygen in Saccharomyces cerevisiae. Microbiol Rev.

[8] DeRisi JL, Iyer VR, Brown PO (1997). Exploring the metabolic and genetic control of gene expression on a genomic scale. Science.

[9] Bourgarel D, Nguyen CC, Bolotin-Fukuhara M (1999). HAP4, the glucose-repressed regulated subunit of the HAP transcriptional complex involved in the fermentation-respiration shift, has a functional homologue in the respiratory yeast Kluyveromyces lactis. Mol Microbiol.

[10] Sybirna K (2005). A new Hansenula polymorpha HAP4 homologue which contains only the N-terminal conserved domain of the protein is fully functional in Saccharomyces cerevisiae. Curr Genet.

[11] Hortschansky P (2007). Interaction of HapX with the CCAAT-binding complex–a novel mechanism of gene regulation by iron. EMBO J.

[12] Hsu PC, Yang CY, Lan CY (2011). Candida albicans Hap43 is a repressor induced under low-iron conditions and is essential for iron-responsive transcriptional regulation and virulence. Eukaryot Cell.

[13] Jung WH (2010). HapX positively and negatively regulates the transcriptional response to iron deprivation in Cryptococcus neoformans. PLoS Pathog.

[14] Krober A (2016). HapX Mediates Iron Homeostasis in the Pathogenic Dermatophyte Arthroderma benhamiae but Is Dispensable for Virulence. PLoS One.

[15] Labbe S, Khan MG, Jacques JF (2013). Iron uptake and regulation in Schizosaccharomyces pombe. Curr Opin Microbiol.

[16] Lopez-Berges MS (2012). HapX-mediated iron homeostasis is essential for rhizosphere competence and virulence of the soilborne pathogen Fusarium oxysporum. Plant Cell.

[17] Schrettl M (2010). HapX-mediated adaption to iron starvation is crucial for virulence of Aspergillus fumigatus. PLoS Pathog.

[18] Singh RP, Prasad HK, Sinha I, Agarwal N, Natarajan K (2011). Cap2-HAP complex is a critical transcriptional regulator that has dual but contrasting roles in regulation of iron homeostasis in Candida albicans. J Biol Chem.

[19] Homann OR, Dea J, Noble SM, Johnson AD (2009). A phenotypic profile of the Candida albicans regulatory network. PLoS Genet.

[20] Gsaller F (2014). The Janus transcription factor HapX controls fungal adaptation to both iron starvation and iron excess. EMBO J.

[21] Merhej J (2015). Yap7 is a transcriptional repressor of nitric oxide oxidase in yeasts, which arose from neofunctionalization after whole genome duplication. Mol Microbiol.

[22] Tanaka A, Kato M, Nagase T, Kobayashi T, Tsukagoshi N (2002). Isolation of genes encoding novel transcription factors which interact with the Hap complex from Aspergillus species. Biochim Biophys Acta.

[23] Petryk N (2014). Functional study of the Hap4-like genes suggests that the key regulators of carbon metabolism HAP4 and oxidative stress response YAP1 in yeast diverged from a common ancestor. PLoS One.

[24] Chen C, Pande K, French SD, Tuch BB, Noble SM (2011). An iron homeostasis regulatory circuit with reciprocal roles in Candida albicans commensalism and pathogenesis. Cell Host Microbe.

[25] Hortschansky P (2015). Deciphering the combinatorial DNA-binding code of the CCAAT-binding complex and the iron-regulatory basic region leucine zipper (bZIP) transcription factor HapX. J Biol Chem.

[26] Kaplan J, McVey Ward D, Crisp RJ, Philpott CC (2006). Iron-dependent metabolic remodeling in S. cerevisiae. Biochim Biophys Acta.

[27] Ihrig J (2010). Iron regulation through the back door: iron-dependent metabolite levels contribute to transcriptional adaptation to iron deprivation in Saccharomyces cerevisiae. Eukaryot Cell.

[28] Philpott CC, Leidgens S, Frey AG (2012). Metabolic remodeling in iron-deficient fungi. Biochim Biophys Acta.

[29] Merhej J (2016). A Network of Paralogous Stress Response Transcription Factors in the Human Pathogen Candida glabrata. Front Microbiol.

[30] Brunke S, Hube B (2013). Two unlike cousins: Candida albicans and C. glabrata infection strategies. Cell Microbiol.

[31] Johnson DC, Cano KE, Kroger EC, McNabb DS (2005). Novel regulatory function for the CCAAT-binding factor in Candida albicans. Eukaryot Cell.

[32] McNabb DS, Tseng KA, Guarente L (1997). The Saccharomyces cerevisiae Hap5p homolog from fission yeast reveals two conserved domains that are essential for assembly of heterotetrameric CCAAT-binding factor. Mol Cell Biol.

[33] Gerwien, F. *et al*. A Novel Hybrid Iron Regulation Network Combines Features from Pathogenic and Nonpathogenic Yeasts. *MBio* **7** (2016). 10.1128/mBio.01782-16 PMC5082906 27795405

[34] Li L, Bagley D, Ward DM, Kaplan J (2008). Yap5 is an iron-responsive transcriptional activator that regulates vacuolar iron storage in yeast. Mol Cell Biol.

[35] Li L, Jia X, Ward DM, Kaplan J (2011). Yap5 protein-regulated transcription of the TYW1 gene protects yeast from high iron toxicity. J Biol Chem.

[36] Pimentel C (2012). The role of the Yap5 transcription factor in remodeling gene expression in response to Fe bioavailability. PLoS One.

[37] Rietzschel N, Pierik AJ, Bill E, Lill R, Muhlenhoff U (2015). The Basic Leucine Zipper Stress Response Regulator Yap5 Senses High-Iron Conditions by Coordination of [2Fe-2S] Clusters. Mol Cell Biol.

[38] Tan K (2008). A systems approach to delineate functions of paralogous transcription factors: role of the Yap family in the DNA damage response. Proc Natl Acad Sci USA.

[39] Avendano A (2005). Swi/SNF-GCN5-dependent chromatin remodelling determines induced expression of GDH3, one of the paralogous genes responsible for ammonium assimilation and glutamate biosynthesis in Saccharomyces cerevisiae. Mol Microbiol.

[40] Dang VD, Bohn C, Bolotin-Fukuhara M, Daignan-Fornier B (1996). The CCAAT box-binding factor stimulates ammonium assimilation in Saccharomyces cerevisiae, defining a new cross-pathway regulation between nitrogen and carbon metabolisms. J Bacteriol.

[41] Dang VD, Valens M, Bolotin-Fukuhara M, Daignan-Fornier B (1996). Cloning of the ASN1 and ASN2 genes encoding asparagine synthetases in Saccharomyces cerevisiae: differential regulation by the CCAAT-box-binding factor. Mol Microbiol.

[42] Hernandez H, Aranda C, Lopez G, Riego L, Gonzalez A (2011). Hap2-3-5-Gln3 determine transcriptional activation of GDH1 and ASN1 under repressive nitrogen conditions in the yeast Saccharomyces cerevisiae. Microbiology.

[43] Hsu PC (2013). Diverse Hap43-independent functions of the Candida albicans CCAAT-binding complex. Eukaryot Cell.

[44] Riego L, Avendano A, DeLuna A, Rodriguez E, Gonzalez A (2002). GDH1 expression is regulated by GLN3, GCN4, and HAP4 under respiratory growth. Biochem Biophys Res Commun.

[45] Gsaller F (2016). Sterol Biosynthesis and Azole Tolerance Is Governed by the Opposing Actions of SrbA and the CCAAT Binding Complex. PLoS Pathog.

[46] Kennedy MA, Barbuch R, Bard M (1999). Transcriptional regulation of the squalene synthase gene (ERG9) in the yeast Saccharomyces cerevisiae. Biochim Biophys Acta.

[47] Thon M (2010). The CCAAT-binding complex coordinates the oxidative stress response in eukaryotes. Nucleic Acids Res.

[48] Sybirna K, Petryk N, Zhou YF, Sibirny A, Bolotin-Fukuhara M (2010). A novel Hansenula polymorpha transcriptional factor HpHAP4-B, able to functionally replace the S. cerevisiae HAP4 gene, contains an additional bZip motif. Yeast.

[49] Kitada K, Yamaguchi E, Arisawa M (1995). Cloning of the Candida glabrata TRP1 and HIS3 genes, and construction of their disruptant strains by sequential integrative transformation. Gene.

[50] Schwarzmuller T (2014). Systematic phenotyping of a large-scale Candida glabrata deletion collection reveals novel antifungal tolerance genes. PLoS Pathog.

[51] Merhej J (2014). bPeaks: a bioinformatics tool to detect transcription factor binding sites from ChIPseq data in yeasts and other organisms with small genomes. Yeast.

[52] Longtine MS (1998). Additional modules for versatile and economical PCR-based gene deletion and modification in Saccharomyces cerevisiae. Yeast.

[53] Puig O (2001). The tandem affinity purification (TAP) method: a general procedure of protein complex purification. Methods.

[54] Lelandais G, Blugeon C, Merhej J (2016). ChIPseq in Yeast Species: From Chromatin Immunoprecipitation to High-Throughput Sequencing and Bioinformatics Data Analyses. Methods Mol Biol.

[55] Thorvaldsdottir H, Robinson JT, Mesirov JP (2013). Integrative Genomics Viewer (IGV): high-performance genomics data visualization and exploration. Brief Bioinform.

[56] Quinlan AR, Hall IM (2010). BEDTools: a flexible suite of utilities for comparing genomic features. Bioinformatics.

[57] Thomas-Chollier M (2012). RSAT peak-motifs: motif analysis in full-size ChIP-seq datasets. Nucleic Acids Res.

[58] Medina-Rivera A (2015). RSAT 2015: Regulatory Sequence Analysis Tools. Nucleic Acids Res.

[59] Csardi, G. & Nepusz, T. The igraph software package for complex network research. *InterJournal Complex Systems*, 1695 (2006).

[60] Inglis DO (2012). The Candida genome database incorporates multiple Candida species: multispecies search and analysis tools with curated gene and protein information for Candida albicans and Candida glabrata. Nucleic Acids Res.

[61] Lemoine S, Combes F, Servant N, Le Crom S (2006). Goulphar: rapid access and expertise for standard two-color microarray normalization methods. BMC Bioinformatics.

[62] Ritchie ME (2015). limma powers differential expression analyses for RNA-sequencing and microarray studies. Nucleic Acids Res.

[63] Oeffinger M (2007). Comprehensive analysis of diverse ribonucleoprotein complexes. Nat Methods.

