# Supplementary material for: The CCAAT-Binding Complex Controls Respiratory Gene Expression and Iron Homeostasis in Candida Glabrata
Source: Sci Rep. 2017 Jun 14;7:3531. doi: 10.1038/s41598-017-03750-5 (PMC5471220; doi:10.1038/s41598-017-03750-5)
Supplement: Supplementary file 1 — supplementary files S1 and S3 to 7 [file 41598_2017_3750_MOESM1_ESM.pdf]

THE CCAAT-BINDING COMPLEX CONTROLS RESPIRATORY GENE EXPRESSION AND IRON HOMEOSTASIS IN CANDIDA GLABRATA.

Antonin Thiébaut[1], Thierry Delaveau[1], Médine Benchouaia[1], Julia Boeri[1], Mathilde Garcia[1], Gaëlle Lelandais[2,3], Frédéric Devaux[1]*.

LEGENDS FOR SUPPLEMENTARY FILES

Supplementary file S1: Main roles reported for the CBC in the model yeast *S. cerevisiae* and in the human pathogen *C. albicans*. See the main text (introduction and discussion) for the corresponding bibliographic references.

Supplementary file S2: (A): list of the promoters which are targeted by Hap5 according to ChIP-seq data. The names of the corresponding gene targets and the presence of a CCAAT motif in the corresponding ChIP-peaks are indicated. (B): Chromosomal positions and quantitative features of the ChIP peaks according to the bpeaks software (ref bpeaks).

Supplementary file S3: ChIP-QPCR analyses of Hap5 binding to *GRX4* promoter as a function of iron concentrations. Hap5-myc tagged strains were grown either in YPD or in YPD supplemented with 2mM of $FeSO_4$ (iron excess) or 0.5 mM of BPS (iron starvation). The untagged strain grown under the same conditions was used as a mock IP control. The values represent the IP/Input ratios of the *GRX4* promoter relative to the enrichment of the *YHB1* promoter (used as an internal control). The experiments were performed twice on biologically independent samples. Error bars hence represent the standard error of the mean.

Supplementary file S4: The relative expression of *COX12* was measured by Q-RTPCR in wild type, *hap5Δ*, *hap4Δ* and *yap5Δ* strains grown in glucose, glycerol or iron excess. The values represent the expression levels of the *COX12* gene relative to *ACT1* (used as an internal control) and to the wild type grown in glucose. The experiments were performed three times on biologically independent samples. Error bars represent the pearson standard deviation. A t-test was performed to compare the mutants to the corresponding wild type for each growth conditions. The results of the test are indicated by the stars as follows *$p<0.05$, **: $p<0.01$, ***: $p<0.001$.

Supplementary file S5: IGV snapshots of the ChIP peaks obtained for the seven targets of Hap5 and Yap5 which contains both a CCAAT and a YRE motif. For each locus, the X-axis represents the genomic sequence of the locus (25 bp scale indicated), the Y-axis is the number of reads obtained in the Hap5-IP (upper row) or the Yap5-IP (lower row) for each nucleotide. The color code for the sequence is: red = T, green = A, blue = C, orange = G. The CCAAT and YRE motifs are indicated by black boxes on the sequence.

Supplementary file S6: Western blot analyses of the wild type and mutants Yap5 protein from total protein extracts. Two independent clones were tested for each strain. The Yap5 protein is fused to 13 c-Myc epitopes, revealed by anti-cMyc primary antibodies and the corresponding band is expected at 65 kDa. Proteins were separated on 10% SDS-Polyacrylamide gel electrophoresis (SDS-PAGE). Proteins were then transferred to Whatman® Protan® BA83 nitrocellulose membrane (GE Healthcare). Immunoblotting of Yap5-myc wild type and mutants protein were performed using 1:10000 mouse IgG Anti-cMyc (Roche) and 1:10000 anti-mouse IgG-HRP (Promega) as primary and secondary antibodies, respectively. The membrane was stripped by boiling 30 minutes in 62.5 mM Tris HCl pH 6.8, SDS 2% and 4 mM DTT. Then, immunoblotting of the ribosomal protein Rpl1, used as a loading control (expected band at 25kDa), was performed using 1:10000 rabbit IgG Anti-Rpl1 (gift from M. Garcia: refer to Delaveau T. et al., Nucleic Acids Research 2016) and 1:15000 anti-rabbit IgG-HRP (Promega) as primary and secondary antibodies, respectively. Detection of the signals was performed using G:BOX Chemi XT4 (Syngene) following incubation with UptiLightTM HRP blot chemiluminescent ECL substrate (Interchim) for Rpl1 or Supersignal West Femto (supplied by Thermofisher scientific) for Yap5-myc.

Supplementary file S7: Western blots analyses of the co-immunoprecipitation experiments using Hap5-Protein A as bait and wild type or mutated versions of Yap5-myc as prey. Immunoblotting was performed with a rabbit IgG-HRP polyclonal antibody (PAP; code Z0113; Dako), which has a high affinity for Protein A. Protein A signal is used as a loading control (INPUT) and as a control for immunoprecipitation (IP) efficiency for the interpretation of results shown in figure 4C. The ladder on the right was copied and pasted from the white light image of the membrane.

Supplementary file S8: Strains used in this study.

Supplementary file S9: Primers used in this study.

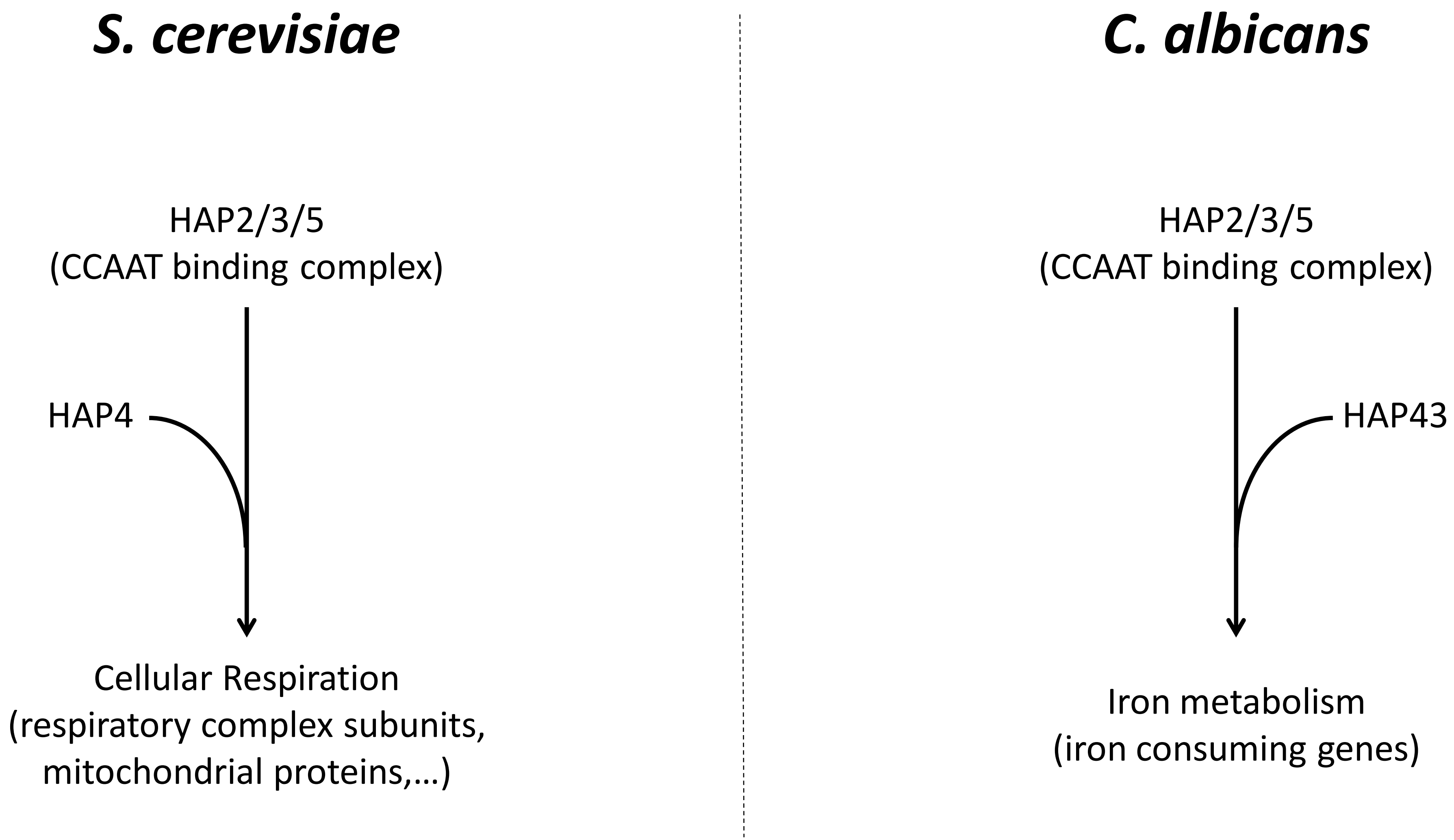

Supplementary file S1

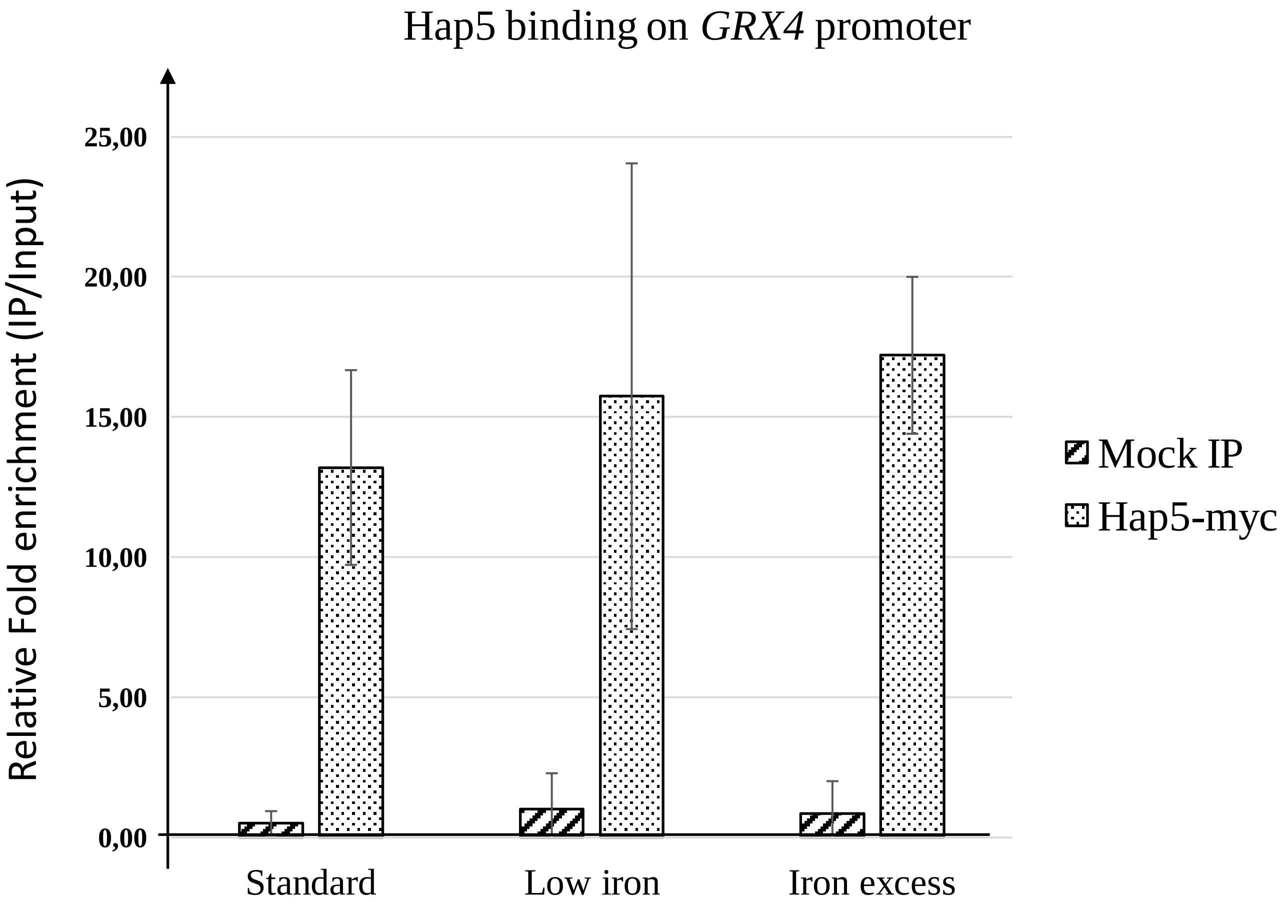

Supplementary file S3

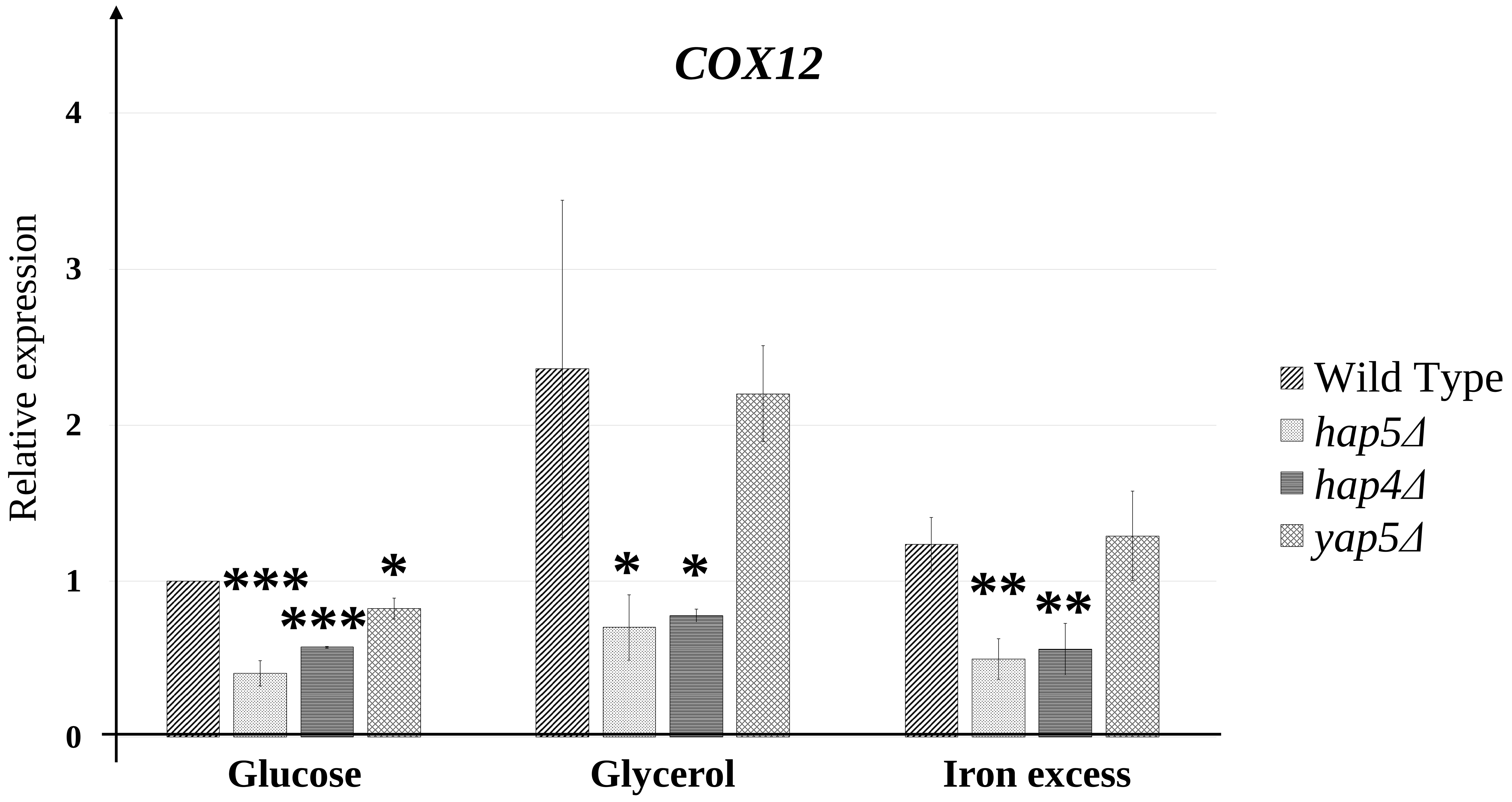

Supplementary file S4

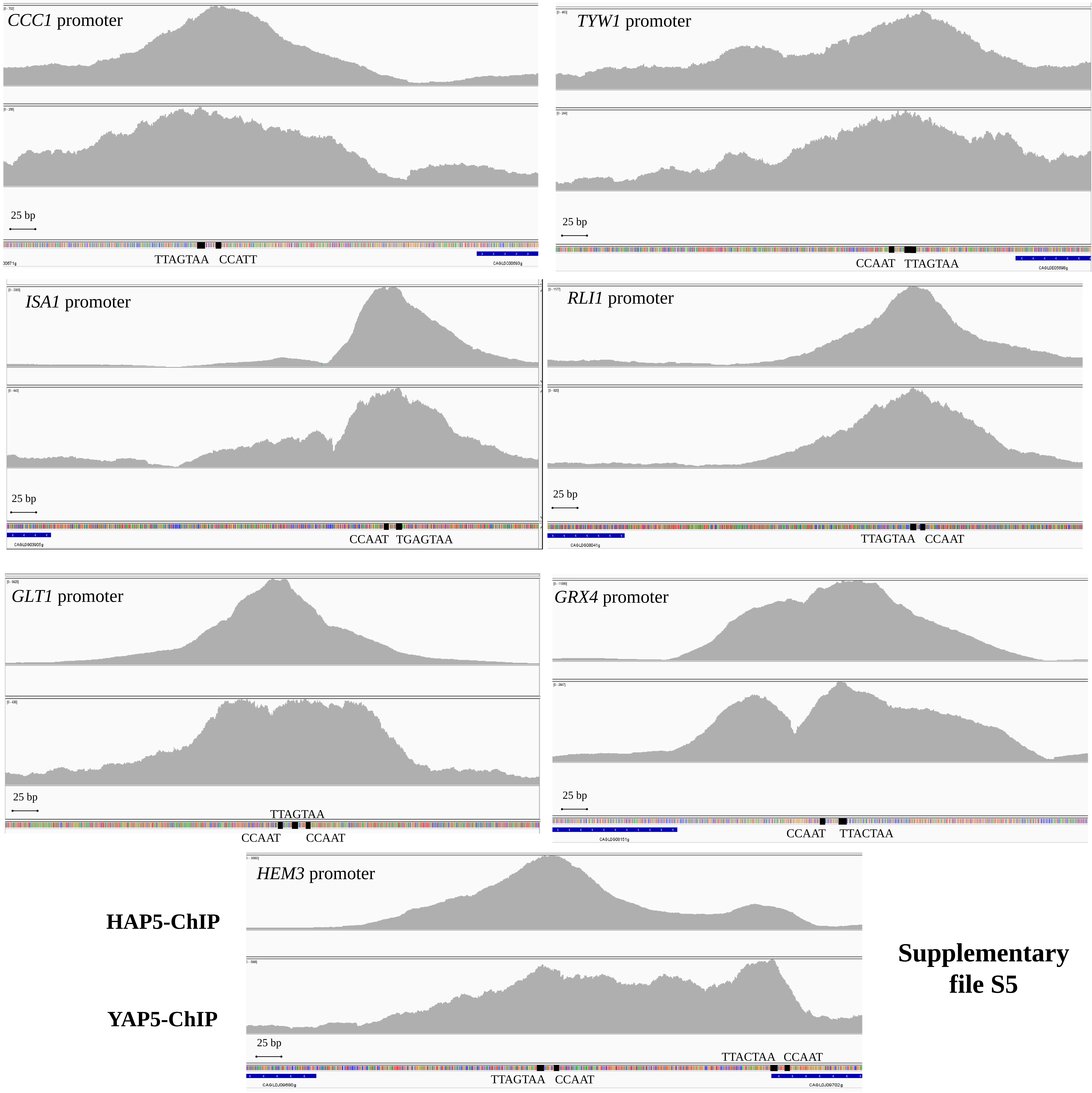

Supplementary file S5

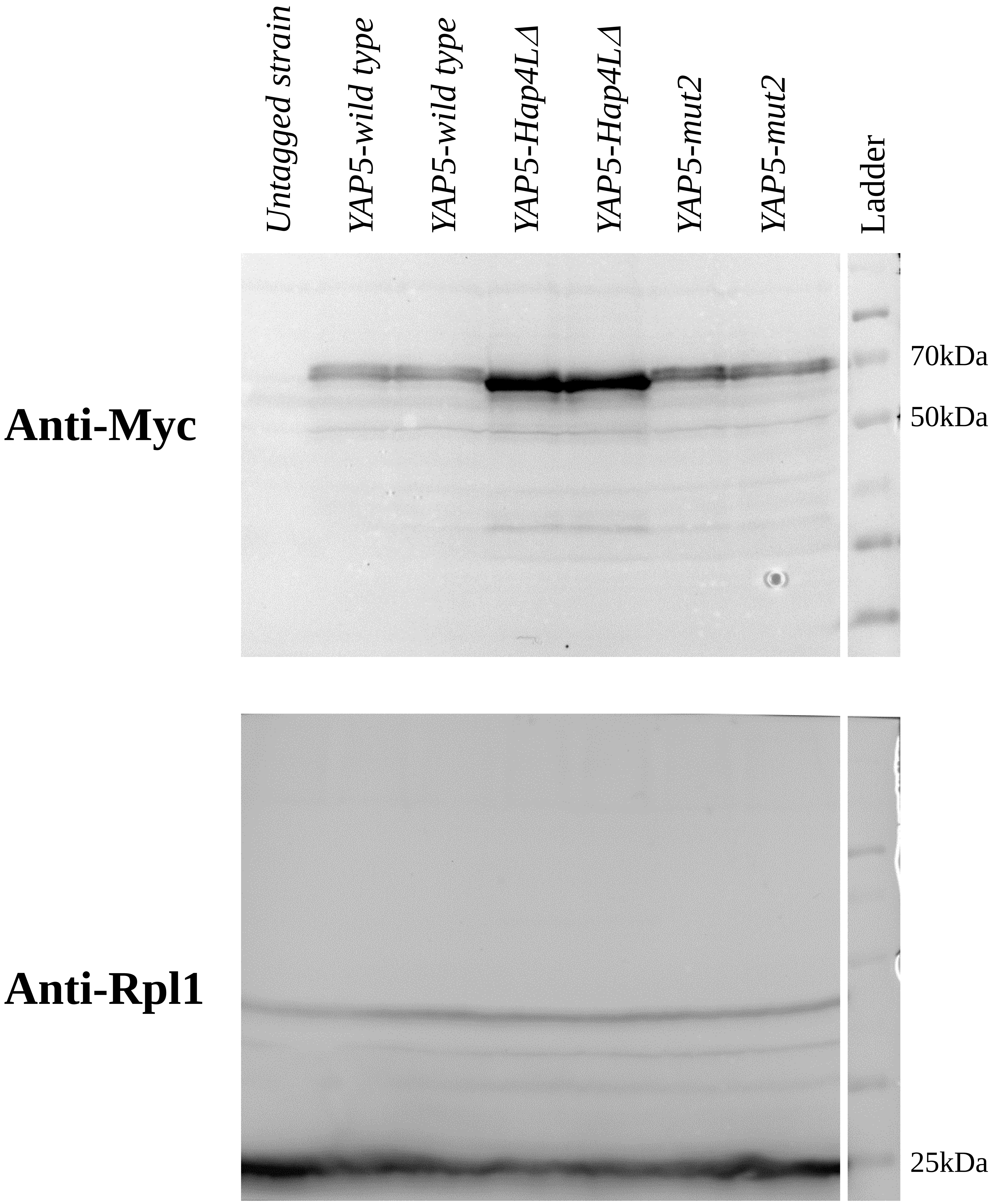

Supplementary file S6

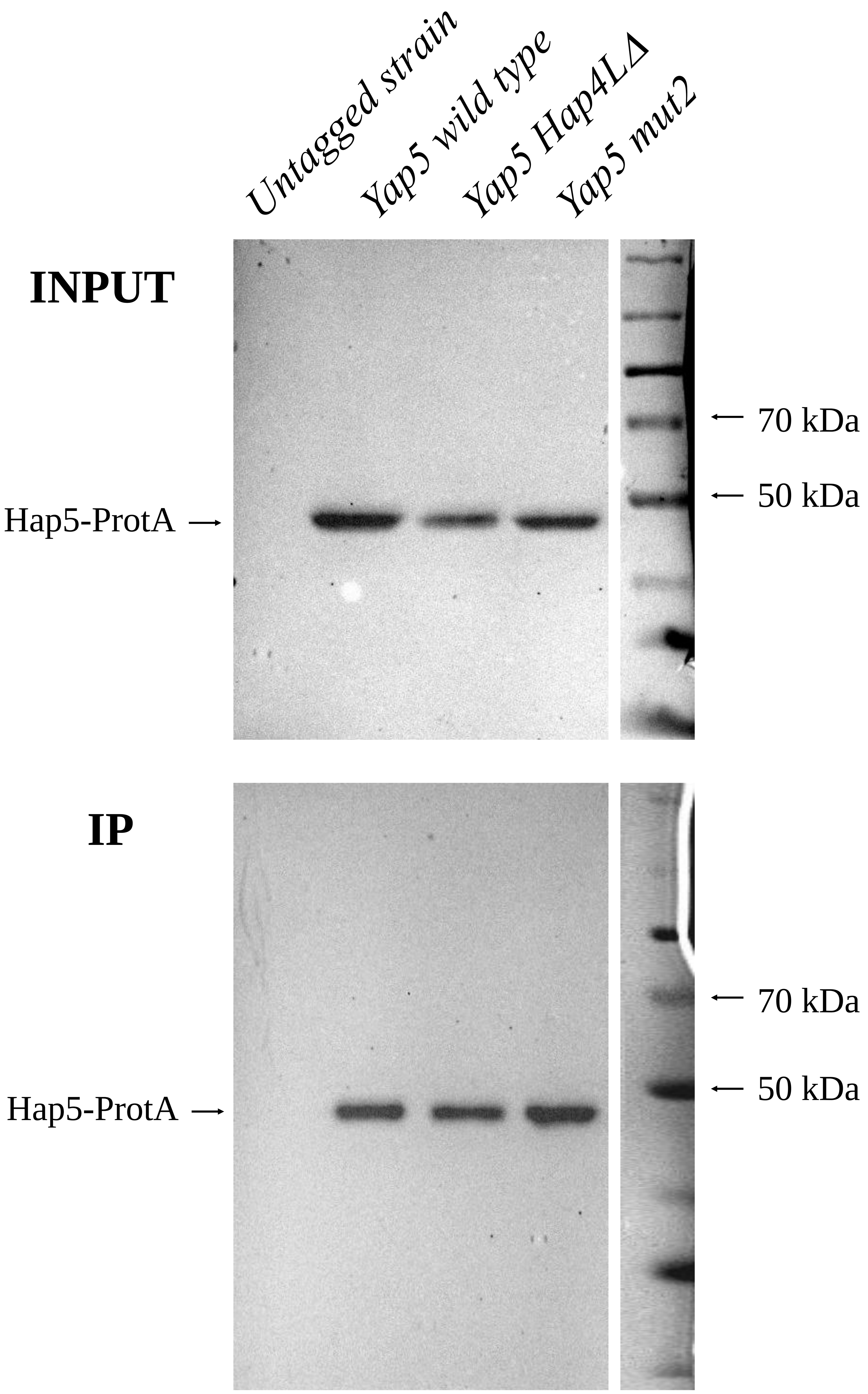

Supplementary file S7

Strain name	Parental strain	Genotype	Origin
ΔHTU	ATCC 2001	his3Δ/trp1Δ/ura3Δ	Kitada et al., 1995
yap5Δ	ΔHTU	CAGL0K08756g::TRP1/his3Δ/trp1Δ/ura3Δ	Merhej et al., 2015
HTL	ATCC 2001	his3Δ/trp1Δ/leu2Δ	Schwartzmuller et al., 2014
hap4Δ	HTL	CAGL0K08624g::NAT/his3Δ/trp1Δ/leu2Δ	Schwartzmuller et al., 2014
hap5Δ	HTL	CAGL0K09900g::NAT/his3Δ/trp1Δ/leu2Δ	Schwartzmuller et al., 2014
Hap5-myc	HTL	CAGL0K09900g-13Myc-HIS5/his3Δ/trp1Δ/leu2Δ	This work
Yap5-Hap4LΔ-myc	HTL	his3Δ/trp1Δ/leu2Δ pGRB2.1-HIS-YAP5-HAP4LΔ-myc	This work
Yap5-mut2-myc	HTL	his3Δ/trp1Δ/leu2Δ pGRB2.1-HIS-YAP5-mut2-myc	This work
Yap5-myc, hap5Δ	hap5D	CAGL0K09900g::NAT/his3Δ/trp1Δ/leu2Δ, pGRB2.1-HIS-YAP5-myc	This work
Hap5-ProtA	HTL	CAGL0K09900g-TAP-TRP1/his3Δ/trp1Δ/leu2Δ	This work
Hap5-ProtA, Yap5-myc	Hap5-ProtA	CAGL0K09900g-TAP-TRP1/his3Δ/trp1Δ/leu2Δ, pGRB2.1-HIS-YAP5-myc	This work
Hap5-ProtA, Yap5-Hap4LΔ-myc	Hap5-ProtA	CAGL0K09900g-TAP-TRP1/his3Δ/trp1Δ/leu2Δ, pGRB2.1-HIS-YAP5-HAP4LΔ-myc	This work
Hap5-ProtA, Yap5-mut2-myc	Hap5-ProtA	CAGL0K09900g-TAP-TRP1/his3Δ/trp1Δ/leu2Δ, pGRB2.1-HIS-YAP5-mut2-myc	This work
Yap5-myc	HTL	his3Δ/trp1Δ/leu2Δ pGRB2.1-HIS-YAP5-myc	This work

Supplementary file S8: Strains used in this study.

Supplementary file S9: Primers used in this study.

NAME	SEQUENCES (5'=>3')	Use
301-PqCgAP6-F	GGGGAATTGTGGGTTCTGTA	Verification of yap5D
300-CgAP6ter-Rev	CCACACTAGTAATGTGGAGAT	Verification of yap5D
880-Hap4TermVerifDel-Rev	GGTGGACAGTACTGGCATTA	Verification of hap4D
854-Hap4ORFSeqVerif-For	GATGAACAAGACGCGCACTCC	Verification of hap4D
699-CgHAP5-Pq-F	CAAGTCGCACTCGCTACC	Verification of Hap5 myc and TAP tagging and of hap5D
700-CgHAP5-Pq-R	CTTGTGCTTCTCGGCAAC	Verification of hap5D
696-CgHAP5-Del-R1	TGCTTCTCGTGCTTCTCTTGCTTCTCTTGCTTCTCTTGTCCTTGCATGTATGTACTTATTCAGGATTACATACTACATAGAATTCGAGCTCGTTTAAAC	Myc Tagging of Hap5
777-CgHap5end-F1	GCAAAAAGCCGATATAGCTGAGGCCCTGCAGATGAGCGATATGTTCGACTTCCTCATCGATATAGTACCTCGTTCCACCATAGGAGCGCAAAACAACTCTTCG	Myc Tagging of Hap5
869-YAP5-delHap4L-for	ATGGCGATGTCCAAGATACATATTAAAGGGAGCCACGAAAGAGCCAAG	deletion of Yap5 Hap4L domain
870-YAP5-delHap4L-rev	CTTGGCTCTTTCGTGGCTCCCTTTAATATGTATCTTGGACATCGCCAT	deletion of Yap5 Hap4L domain
871-YAP5-MutS33P/K34E-for	AAGATACATATTCCAGAAAAGTGGAAGCTG	mutagenesis of Yap5 Hap4L domain S33P/K34E
872-YAP5-MutS33P/K34E-rev	CAGCTTCCACTTTTCTGGAATATGTATCTT	mutagenesis of Yap5 Hap4L domain S33P/K34E
875-YAP5-VerifSeqMut-for	GCTGACTGCTCTGGGATCAATG	sequencing of mutagenized plasmids
698-CgHAP5-ter-R	GTACAGGACATCCTTGCTTC	Verification of Hap5 myc tagging
329-HIS5-For	GCAAACCAAAAGGGAGAACA	Verification of Hap5 myc tagging
330-HIS5-Rev	GGACAATTCCCCAACCTTTT	Verification of Hap5 myc tagging
464-CAGL0G08151gPr-F	AACCAGCAAACCAGGAACAC	ChIP-QPCR GRX4
465-CAGL0G08151gPr-R	TGCATTACTAATCGCCACCA	ChIP-QPCR GRX4
466-Pq-CAGL0G08151g-F	TCAGATTCGGGTTCTTCGAC	RT-QPCR GRX4
467-Pq-CAGL0G08151g-R	TAGGCCACCTTGGAACTCAC	RT-QPCR GRX4
883-CgCOX12-Q-for	GACACTGCTGGCAAGCATATG	RT-QPCR COX12
884-CgCOX12-Q-rev	CCATTCGACAGGACATAGAG	RT-QPCR COX12
885-CgATP2-Q-for	CCATTCGCTGTCGCTGAAG	RT-QPCR ATP2
886-CgATP2-Q-rev	GCAACGACATCTTCAATACC	RT-QPCR ATP2
462-CgYHB1Prom-F	ACCTCTCGGGACAATGAACA	control for ChIP-QPCR
463-CgYHB1Prom-R	TCTGTGTTTCTTTTTGCGTTG	control for ChIP-QPCR
Q-CgACT1-For	GCTCCAGAAGCTTTGTTCCACCCAT	control for RT-QPCR
Q-CgACT1-Rev	CATCACACTTCATGATGGAGTTGTA	control for RT-QPCR
923-CgHAP5-ProtA-F	CTGCAGATGAGCGATATGTTCGACTTCCTCATCGATATAGTACCTCGTTCCACCATAGGAGCGCAAAACAACTCTTCGTCCATGGAAAAGAGAAG	TAP-tagging of Hap5
924-CgHAP5-ProtA-R	TCTCGTGCTTCTCTTGCTTCTCTTGCTTCTCTTGTCCTTGCATGTATGTACTTATTCAGGATTACATACTACATATACGACTCACTATAGGG	TAP-tagging of Hap5
914-TapGwenael-R	CGTCTACTTTCGGCGCCTG	verification of TAP-tagging
